# Giant liver tumor causing dyspnea upon exertion

**DOI:** 10.1002/ccr3.1586

**Published:** 2018-05-15

**Authors:** Nikolaos Machairas, Zoe Garoufalia, Georgios C. Sotiropoulos

**Affiliations:** ^1^ The 2nd Department of Propaedeutic Surgery National and Kapodistrian University of Athens Medical School Athens Greece

**Keywords:** diaphragm elevation, dyspnea, giant, liver tumor

## Abstract

Asymptomatic elevation of the right hemidiaphragm should always raise suspicion of a silent hepatic tumor. Prompt multimodality imaging plays a critical role in the identification of this entity; high clinical suspicion is the key element for diagnosis of a possible hepatic tumor.

## 1 CASE PRESENTATION

**Figure 1:**
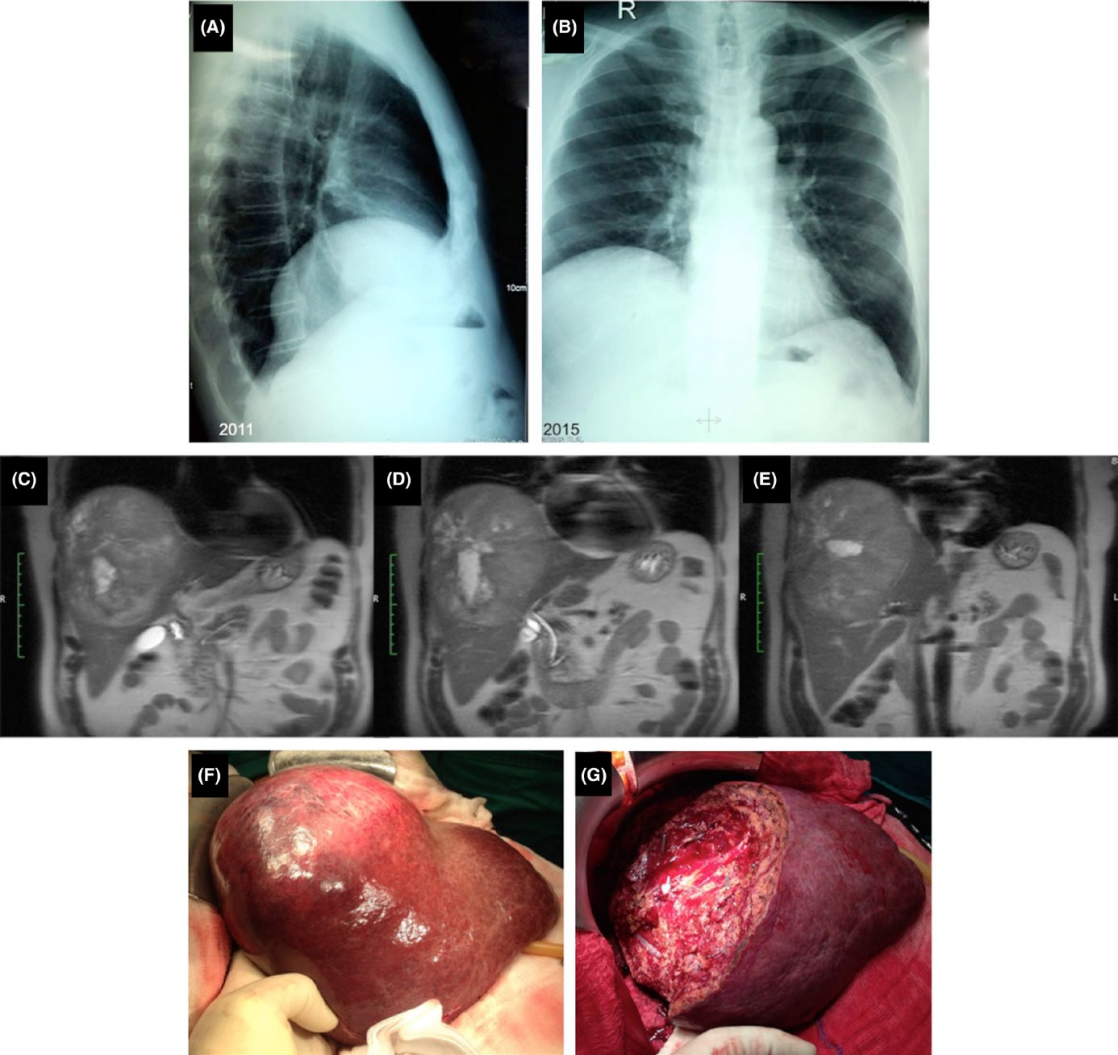
A, Previous thorax X‐ray of the patient (2011). B, Thorax X‐ray of the patient at presentation (2015). C‐E,Magnetic resonance imaging of the giant liver lesion. F, G, Intraoperative images of the central liver lesion prior (F) and after resection (G)

A 73‐year‐old overweight male patient complained about mild right subcostal abdominal pain and deteriorating dyspnea upon exertion. The patient had previously undergone multiple thorax X‐rays and was diagnosed with an asymptomatic elevation of his right hemidiaphragm (Figure 1A). A new thorax X‐ray showed persistent right‐sided hemidiaphragmatic elevation (Figure 1B). Physical examination revealed decreased to eliminated right lung base breath sounds. Percussion in this area was evident for a solid mass. Lung or subdiaphragmatic liver tumor was part of the differential diagnosis. Abdominal ultrasound and cross‐sectional imaging (thorax‐CT, abdominal MRI) revealed a giant subdiaphragmatic hepatic lesion (16 × 12 × 9 cm), centrally located in the liver (segments IVa, VIII, VII, and partially IVb and V) (Figures 1C‐E). Serological examinations were negative for viral hepatitis. Alcohol intake was referred to as light to moderate. The suspicion of hepatocellular adenoma was raised. The patient was admitted to our hospital and underwent an atypical central hepatic resection (Figures 1F,G). His postoperative course was uneventful and he was discharged on 8th postoperative day. Histological examination showed a well‐differentiated HCC, (pT1) arising from nonalcoholic steatohepatitis, resected in clear margin (R0). The patient remains in excellent general condition and recurrence‐free 38 months postoperatively.

Progressive dyspnea can be a frequent finding in geriatric patients due to several causes.^1^ On the other hand, dyspnea as a primary symptom due to hemidiaphragm elevation in the context of an underlying liver tumor is extremely rare.^2^ Moreover, symptomatic elevation of the right hemidiaphragm should always raise suspicion of a silent hepatic tumor.

## AUTHORSHIP

NM and GCS: designed and conceived the study. GCS: acquired the data. NM and ZG: wrote the paper. GCS and NM: analyzed and interpreted the data.

## CONFLICT OF INTEREST

None declared.
